# The effectiveness and safety of extracorporeal shock wave lithotripsy for the management of kidney stones: A protocol of systematic review and meta-analysis

**DOI:** 10.1097/MD.0000000000021910

**Published:** 2020-09-18

**Authors:** Lin Cao, Yun-qi Wang, Tianqiang Yu, Yanli Sun, Jia He, Yun Zhong, Xianming Li, Xianjun Sun

**Affiliations:** a Department of Surgery, Zhejiang Veteran Hospital; b Department of Surgery, Jiaxing Maternal and Child Health Hospital, Jiaxing City, Zhejiang Province, China.

**Keywords:** extracorporeal shockwave lithotripsy (ESWL), kidney stones (KS), meta-analysis

## Abstract

**Background:**

Extracorporeal shockwave lithotripsy (ESWL) has gained worldwide popularity as one of the most commonly used minimally invasive management of urinary tract stones. The objective of this study was to evaluate the efficacy and safety of ESWL for patients with kidney stones (KS).

**Materials and methods:**

This protocol established in this study has been reported following the Preferred Reporting Items for Systematic Review and Meta-Analysis Protocols. Web of Science, PubMed, Embase, Cochrane Library, China Biomedical Literature Database (CBM), China Knowledge Network Database (CNKI), Chinese Scientific Journal Database (VIP), and Wan Fang Database were searched for case–control studies in ESWL treating patients with KS until July 1, 2020. We will use a combination of Medical Subject Heading and free-text terms with various synonyms to search based on the Eligibility criteria. Two investigators independently reviewed the included studies and extracted relevant data. The relative risk (RR) and 95% confidence intervals (CIs) were used as effect estimate. *I*^2^ test, substantial heterogeneity, sensitivity analysis, and publication bias assessment will be performed accordingly. Stata 14.0 and Review Manger 5.3 are used for a meta-analysis.

**Results:**

The results will be published in a peer-reviewed journal.

**Conclusion:**

The results of this review will be widely disseminated through peer-reviewed publications and conference presentations. This evidence may also provide helpful evidence of the efficacy and safety of ESWL treating patients with KS.

**PROSPERO registration number:**

CRD42019157243

## 1 Introduction

Urolithiasis is considered as one of the major health care problems because of its high prevalence, incidence, and recurrence.^[1]^ Extracorporeal shockwave lithotripsy (ESWL) was first used to treat renal calculi in 1980 and since then it has gained worldwide popularity as one of the most commonly used minimally invasive management of urinary tract stones.^[2]^ Initially, ESWL was used to treat upper ureteric and renal stones.^[3,4]^ Later, it became one of the treatment options for distal ureteric stones as it lacks undesirable side effects, requires no anesthesia, low cost yet very powerful and safe.^[5,6]^Patients’ position for distal ureteric stones during ESWL is still a matter of debate as there is lack of published articles about this issue in the current literature. Most urologists preferred prone ESWL with the head therapy in contact with the patient's anterior abdomen as the bony pelvis act as a barrier against transmission of the shockwaves to the lower ureter. It was reported to be a safe and effective approach of distal ureteric stones management.^[7,8]^

Kidney stones (KS), also known as nephrolithiasis, is a very common urological disease.^[9,10]^ It has been estimated that its prevalence rates are up to 14.8% and increasing, and its recurrence rates are up to 50% within the subsequent 5 to 10 years after the first episode.^[11,12]^ If it cannot be treated effectively, it can cause significant morbidity, and can seriously impact quality of life in patients with KS.^[13]^ Risk factors including obesity, diabetes mellitus, hypertension and metabolic syndrome contribute to the KS formation.^[14,15]^ A variety of managements for KS are available, such as acupuncture, herbal medicine, surgery, dietary supplementation, oral medicine, and extracorporeal shock wave lithotripsy (ESWL). A numerous studies have reported that ESWL can effectively treat patients with KS.^[16]^

Therefore, the objective of this study was to assess the efficacy and safety of ESWL for patients with KS and perform a meta-analysis based on all available prospective studies.

## 2 Study aim

The aim of our study is to provide helpful evidence of the efficacy and safety of ESWL for patients with KS. A better understanding of whether ESWL has better advantage in the patients with KS.

## 3 Materials and methods

The protocol of our meta-analysis followed the guideline of the Preferred Reporting Items for Systematic Review and Meta-Analysis Protocols (PRISMA-P) recommendations.^[17]^ It has been registered with International Prospective Register of Systematic Reviews (PROSPERO) as CRD42019157243 (https://www.crd.york.ac.uk/prospero/display_record.php?ID=CRD42019157243).

### 3.1 Eligibility criteria

#### 3.1.1 Types of studies

Prospective cohort, retrospective cohort, or case–control studies of the efficacy and safety of ESWL for patients with KS, will be included to pool and review in this study.

#### 3.1.2 Types of participants and interventions

In this study, we will include the patients who were diagnosed with ESWL with no restrictions on country, ethnic background, sex, age, or economic status.

#### 3.1.3 Types of outcome

Outcomes will include overall stone-free rate, mean stone size (millimeter), pain intensity, urinary biochemical variables, mean hospital stay (day), quality of life, and adverse events and associated 95% confidence intervals (CIs).

### 3.2 Search strategy

Web of Science, PubMed, Embase, Cochrane Library, China Biomedical Literature Database (CBM), China Knowledge Network Database (CNKI), Chinese Scientific Journal Database (VIP), and Wan Fang Database were searched for cohort and case–control studies in cases until July 1, 2020. The MeSH search and text word will be used with the terms related to extracorporeal shockwave lithotripsy and kidney stones. To perform a comprehensive and focused search, experienced systematic review researchers will be invited to develop a search strategy. An example of search strategy for PubMed database shown in Table 1 will be modified and used for the other databases. The reference lists of all relevant studies will be searched for additional relevant studies not retrieved from the electronic database search.

**Table 1:**
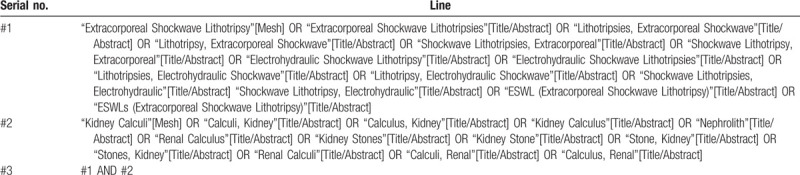
Searching strategy in PubMed.

### 3.3 Study selection

**Figure 1:**
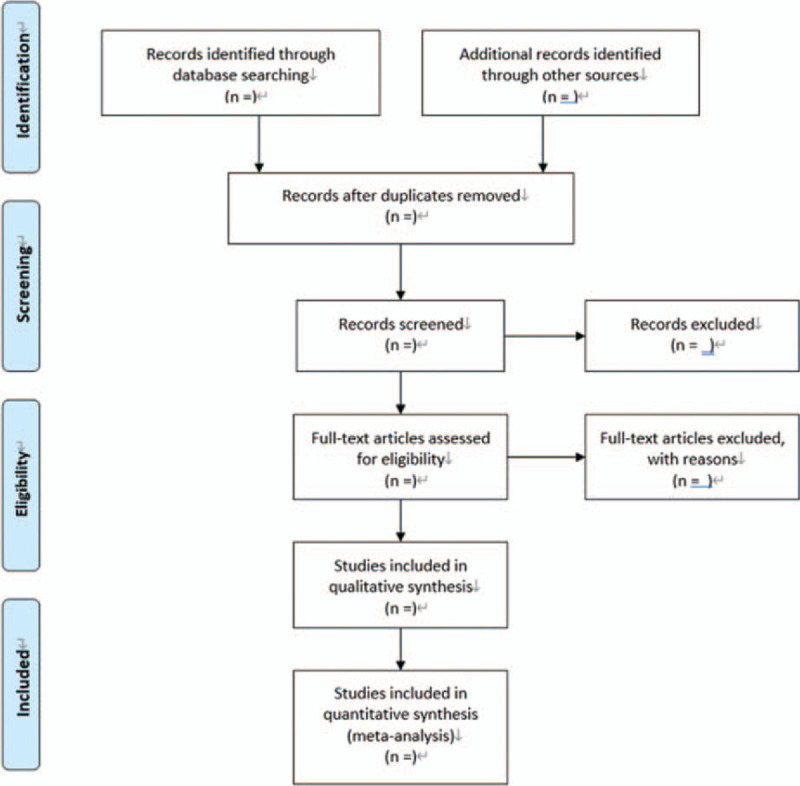
Flow diagram: selection process for the studies.

All initial records from 4 electronic databases will be imported into the web-based systematic review Rayyan software.^[18]^ First, the titles and abstracts of records will be reviewed independently by 2 reviewers to identify potential trials according to eligibility criteria. Then, full text of all potentially relevant trials will be downloaded to make sure eligible trials. Any conflict will be resolved by discussion. A flow diagram (Fig. 1) will be used to describe the selection process of eligible articles.

### 3.4 Data extraction and management

The studies retrieved during the searches will be screened for relevance, and those identified as being potentially eligible will be fully assessed against the inclusion/exclusion criteria, and accepted or rejected, as appropriate. The following information will then be independently extracted by 2 researchers using a predesigned and standardized sheet: first author, publication year, location, race, age, sex, disease course and duration, diagnostic criteria, eligibility criteria, sample size, study setting, methods of randomization, blinding, and concealment, treatment details, all outcome measurements, safety, and funding information.

### 3.5 Risk of bias of individual study and quality assessment

We will apply the Cochrane systematic review methods^[19]^ to evaluate the quality of the ultimate included studies. The studies will be graded based on: random sequence generation, allocation concealment, blinding, completeness of outcome data, selective outcome reporting, other bias. If we cannot get the information to do the assessment from the articles, we will connect the corresponding author with telephone to get the true situation. Two of the authors will do this work independently, and if there is any disagreement taking place, the arbiter will do the final judge.

### 3.6 Data analyses

The effect estimate of interest will be the relative risk. Statistical analyses will be performed using Review Manager 5.3 statistical software and Stata 14.0 software. The outcomes will be presented as the relative risk, mean difference or standardized mean difference and its 95% CI. The statistical significance will be assessed for *P* < .05, and moderate to high levels of heterogeneity will be considered for *I*^2^ > 50%.^[20]^ A fixed-effects model will be used if no statistical heterogeneity across the studies; otherwise, the random-effects model will be considered.

### 3.7 Publication bias

If included studies were >10, funnel plot will be used to identify the possible publication bias. Additionally, Egg regression and Begg tests will be utilized to detect the funnel plot asymmetry.^[21]^

## 4 Discussion

A number of studies have reported that patients with KS can achieve encouraging benefits after ESWL treatment. However, their results are still not consistent. Although a recent associated systematic review has been published,^[22]^ there is still several high-quality RCTs addressing this issue after that.^[20,21]^ This systematic review and meta-analysis will evaluate the effectiveness and safety of ESWL treating patients with KS. The results of this review will be widely disseminated through peer-reviewed publications and conference presentations. This evidence may also provide helpful evidence of whether ESWL would have better curative effect for patients with KS.

## Author contributions

Conceptualization: Lin Cao, Xianjun Sun; Acquisition: Lin Cao, Xianjun Sun, Tianqiang Yu; Registration: Yun-qi Wang; Methodology: Jia He, Yun Zhong, Xianming Li; Project administration: Lin Cao, Xianjun Sun; Writing and original draft: Lin Cao, Xianjun Sun, Tianqiang Yu, Yanli Sun, Jia He, Yun Zhong, Xianming Li.

## Abbreviations

Abbreviations: CIs = confidence intervals, ESWL = Extracorporeal shockwave lithotripsy, KS = kidney stones, PRISMA-P = Preferred Reporting Items for Systematic Review and Meta-Analysis Protocols, RR = relative risk.
